# Fibroblast Growth Factor Signaling in Development and Disease

**DOI:** 10.3390/ijms24119734

**Published:** 2023-06-04

**Authors:** Seth Bollenbecker, Jarrod W. Barnes, Stefanie Krick

**Affiliations:** Division of Pulmonary and Critical Care Medicine, Department of Medicine, The University of Alabama at Birmingham, Birmingham, AL 35233, USA; setheb@uab.edu (S.B.); jbarnes@uabmc.edu (J.W.B.)

Fibroblast growth factors (FGFs) and their cognate receptors (FGFRs) are important biological molecules with a wide array of pleiotropic functions. Consisting of 22 distinct mammalian FGF ligands and four highly conserved transmembrane receptor tyrosine kinases (FGFR1–4), this vast molecular network plays a role in nearly every organ of the body and many other biological processes throughout the course of life, including development, metabolism, tissue repair, and angiogenesis [1]. Both FGFs and FGFRs have been implicated in numerous pathological conditions, including developmental and metabolic disorders, neurological disease, and cancer. Because of the broad implications of FGFs, understanding the molecular mechanisms underlying the FGF-FGFR interaction and their downstream signaling events is critical for the development of new therapeutic strategies surrounding disease treatment. Although our understanding related to altered FGF signaling on clinical outcomes and cellular function has advanced immensely, the breadth of the biological impacts that these pathways possess leaves many scientific questions unanswered thus far. This Special Issue, entitled “Fibroblast Growth Factor Signaling in Development and Disease”, within the *International Journal of Molecular Sciences* encompasses six contributions: four original articles and two reviews highlighting novel advancements to understanding FGF signaling pathways and how they modulate cellular processes in health and disease.

The articles in this Special Issue largely focus on two hormones, FGF21 and FGF23, which play important roles in regulating metabolic and skeletal functions in the body. FGF21 is produced primarily by the liver in response to various metabolic stresses and has been shown to improve insulin sensitivity and lipid profiles in animal models [2]. It functions as a regulator of glucose and lipid metabolism, making it a relevant target for metabolic disorders such as type 2 diabetes and obesity [3]. FGF23, on the other hand, is produced mainly in the bone and regulates phosphate homeostasis in the body. By decreasing renal phosphate reabsorption and suppressing the production of active vitamin D, a known regulator of intestinal phosphate absorption, FGF23 plays a key role in mediating mineral metabolism [4]. In individuals with elevated FGF23 levels, such as those with chronic kidney disease (CKD), FGF23 may contribute to systemic inflammation by activating immune cells and inducing the production of pro-inflammatory cytokines [5,6,7].

Czaya et al. [5] thoroughly reviewed the role of FGF23 as a risk factor for chronic inflammation and iron dysregulation. As detailed in the paper, the authors describe a perpetuating cycle of disease-driven systemic inflammation that induces FGF23 production. This can, in turn, cause the liver, lungs, and/or peritoneal macrophages to increase cytokine secretion, thus restarting the inflammatory cycle. Linking FGF23 and anemia was another main focus of this review. Described as a direct action of FGF23, the hormone can suppress erythropoietin (EPO) secretion from the kidney, which ultimately prevents erythroid progenitor differentiation. More indirectly, excess serum FGF23 levels can stimulate the release of pro-inflammatory cytokines, such as interleukin (IL)-6 and IL-1β, which promote hepcidin production in the liver. Hepcidin, a hormonal regulator of iron, blocks the absorption of iron in the small intestine and binds ferroportin, causing its degradation and a subsequent reduction in the amount of iron released into the bloodstream from cells. The authors conclude their review by suggesting some future research opportunities to aid in elucidating these inflammatory and anemic actions, such as examining disrupted FGF23 processing events during its cleavage into C- and N-terminal fragments.

To discover new FGF23-mediated cardiac pathologies, Böckmann et al. [8] evaluated left ventricular hypertrophy (LVH) and fibrosis in 5/6 nephrectomized rats. The researchers found that FGF23 activates the renin–angiotensin–aldosterone system (RAAS) locally in the heart, which is responsible for regulating blood pressure and electrolyte balance in the body. Specifically, FGF23 increased the expression of renin, a kidney-derived hormone that converts angiotensinogen into angiotensin I, which is subsequently converted into angiotensin II by angiotensin-converting enzyme (ACE). Angiotensin II is a potent vasoconstrictor that increases blood pressure and promotes inflammation and fibrosis in various organs, including the heart [9]. FGF23-induced activation of the local RAAS in the heart was shown by the authors to increase the production of angiotensin II and promote cardiac hypertrophy and fibrosis. Furthermore, FGF23-induced activation of the local RAAS was found to be independent of the canonical systemic RAAS activation process, which is commonly associated with hypertension and cardiovascular disease. This suggests that the FGF23-mediated activation of the local RAAS may be a novel therapeutic target for preventing or treating cardiac hypertrophy and fibrosis in patients with CKD or other conditions associated with FGF23 dysregulation.

Silva et al. [10] also took an interest in CKD-associated heart disease and proposed the use of FGF23 and α-Klotho as potential biomarkers. a-Klotho, which is a coreceptor for FGF23 binding to FGFR1, has previously been shown to inhibit cardiac hypertrophy [11]. With an enrollment of 107 patients with diabetes and stage 2–3 CKD, individuals were followed prospectively and divided into three groups based on LV mass index and relative wall thickness to assess the traditional four-pattern classification of cardiac hypertrophy. While this four-pattern classification is very sensitive as an indicator of cardiac hypertrophy [12], the group noted the non-specificity of the classification and a possible need for accompanying biomarkers to determine the etiology of the remodeling taking place within the heart. This study found that both α-Klotho and FGF23 were independently regulated and associated with concentric hypertrophy in CKD patients, with low α-Klotho and elevated FGF23 levels posing the greatest risk to altered cardiac physiology. Along with being identified as independent variables of hospitalization due to cardiovascular events, FGF23 levels ≥ 168 RU/mL and α-Klotho < 313 pg/mL were associated with increased fatal cardiovascular events as well. Findings from Silva et al. aimed to fill the gap of the lack of sensitive, specific, and early prognostic biomarkers for CKD and its associated risk of cardiovascular morbidity and mortality. The group notes that future studies are needed to evaluate the effects of abnormal α-Klotho and FGF23 levels on left ventricular diastolic function and how these contribute to heart failure.

FGF23 not only plays a role as a potential biomarker in cardiovascular disease but also in chronic pulmonary disease [13]. Gulati et al. [14] explored the relationship between chronic obstructive pulmonary disease (COPD) exacerbations and circulating FGF23 levels across a cohort of 70 stable COPD patients. Individuals were grouped into either zero, one, or two or more acute exacerbation events in the previous year. Exacerbations, a severe worsening of respiratory symptoms, are associated with an acceleration of lung function decline and airway inflammation [15]. The frequency of these exacerbations affects hospitalization status and mortality through cardiovascular complications and the worsening of other related comorbidities. This four-month cross-sectional study examined retrospective and prospective exacerbation frequency, based on their timing in relation to initial plasma sampling. FGF23 was found to be associated with a frequent exacerbator phenotype, both retrospectively and prospectively, and was independently associated with the use of supplemental oxygen. Stemming from these findings, the authors note that FGF23 may be an appropriate biomarker for the frequent exacerbator phenotype in COPD patients and may be involved in the pathogenesis of exacerbations through its pro-inflammatory effects. The study also highlights the need for further research to elucidate the mechanisms underlying the association between FGF23 levels and COPD exacerbations, which may inform the potential therapeutic implications of targeting FGF23 in COPD patients.

Switching focus to another FGF ligand, Martínez-Garza et al. [16] reviewed the adaptive response of FGF21 to aberrations in nutritional homeostasis. Beginning with the basic biology of FGF21, the authors touch on its production which stems primarily from the liver, and induction in response to various nutritional challenges such as fasting, overfeeding, protein restriction, a high carbohydrate diet, or a ketogenic diet. Secretion also occurs in the skeletal muscle, white and brown adipose tissue, intestine, heart, kidneys, and pancreas. FGF21 exerts its effects by binding to FGF receptors, which are expressed in many cell types, including adipocytes, hepatocytes, and pancreatic β-cells, which are relevant to this review’s focus on metabolism [17]. During fasting, FGF21 promotes lipolysis in adipose tissue, leading to the release of fatty acids that can be utilized as a source of energy. FGF21 also promotes gluconeogenesis, the production of glucose from non-carbohydrate sources such as amino and fatty acids, in the liver to maintain blood glucose levels during periods of fasting. The review further discusses the role of FGF21 in regulating energy expenditure and body weight. FGF21 has been shown to increase energy expenditure in mice and humans, leading to a reduction in body mass and adiposity [18]. Additionally, it has also been found to promote the browning of white adipose tissue, which can increase thermogenesis and energy expenditure as well. This review summarized the findings surrounding the use of FGF21 in obesity and metabolic disorder treatments. Although elevated in pathological conditions such as liver disease and insulin resistance, the mechanisms by which FGF21 expression is induced and subsequently activated in metabolic pathways are poorly understood. The authors highlighted a positive correlation between FGF21 and insulin levels and indicated that the growth factor may be secreted post-carbohydrate intake. Unfortunately, the use of FGF21 in patients with type 2 diabetes did not improve body weight or blood sugar levels, although dyslipidemia and steatosis were both ameliorated. Understanding these mechanisms more thoroughly may lead to new opportunities for weight loss and glycemic control in the future; however, additional research is required for these advances.

Lastly, Scholle et al. [19] investigated the role of FGF21 as a biomarker in patients with carnitine palmitoyltransferase (CPT) II deficiency. As previously highlighted, FGF21 plays many roles in metabolism, and the authors discussed its use as a biomarker for mitochondrial diseases. CPT I and II are important players in the transport of long-chain fatty acids through the mitochondrial inner membrane. CPT II deficiency is the primary lipid metabolism defect in skeletal muscle. Mice with a liver- or adipose-specific knockout of CPT II have increased serum FGF21 levels and *Fgf21* mRNA expression levels, respectively [20,21]. This provided the impetus for the investigators to study FGF21 levels in CPT II-deficient patients, which is an area not previously studied. Furthermore, the authors noted that the expression of CPT II genes and citrate synthase activity was increased after treating myoblasts with FGF21. In humans, CPT II deficiency in muscle is marked by myalgia and myoglobinuria, which are exacerbated after strenuous exercise, fasting, and exposure to extreme temperatures. This study included 13 patients with genetically confirmed CPT II deficiency and 50 age- and sex-matched healthy controls with no clinical or molecular evidence of mitochondrial disorders. Serum FGF21 levels were measured in both groups using an enzyme-linked immunosorbent assay (ELISA). The results showed that there was no significant difference in FGF21 levels between patients with CPT II deficiency (mean = 66.2 pg/mL) and healthy controls (mean = 68.5 pg/mL). Additionally, no significant correlation was found between serum FGF21 concentrations and the frequency of myalgia attacks up to one-year post-sample collection. Though differences were found between mouse knockout models and patients deficient in CPT II with respect to FGF21 levels, the authors noted that the complete loss of CPT II activity and reduction in CPT I and CPT II protein concentrations were not observed in humans, which may explain the discrepancies. The small sample size was also noted as a limitation of this study. However, human muscle CPT II deficiency is a rare disease; therefore, the cohort size was quite large compared to other studies in this area. Based on the data presented in this study, FGF21 does not seem to be reliable as a biomarker to diagnose CPT II deficiency.

Given the broad range of topics covered in just a few articles for this Special Issue, the widespread involvement of FGFs in development and disease is abundantly clear. The use of FGFs as biomarkers in disease identification, the modulation of associated signaling pathways, and even the inhibition of the ligands or receptors themselves is a quickly evolving field that may lead to many therapeutic discoveries. These studies and those that build on them will contribute greatly to our understanding of FGF signaling in health and disease over the coming years.
